# The Tri-State Dental Meeting

**Published:** 1895-07

**Authors:** 


### The Tri-State Dental Meeting.

The joint meeting of the State Societies of Ohio, Michigan and Indiana was held as previously arranged and announced, in Detroit, June 18, 19 and 20, in the Dental College, in Detroit. The meeting was a success in all respect, far beyond the most ardant expectations. The attendance was much larger than was anticipated by any one, even the Committee, whose business it was to correspond and estimate the probable attendance, were greatly surprised at the numbers present. Not only was there a large number of the membership of the three State Societies present, but also a large number of non-members from the respective States, but from other States as well, viz: Illinois, Missouri, Kentucky, Pennsylvania and New York; in all about three hundred and fifty.

The papers read, of which there were a goodly number, were upon very interesting subjects, and well prepared, and fully discussed. Interesting clinics were conducted on two afternoons, in which many valuable practicable matters were presented. Each of the State Societies held their respective meetings for the transaction of such routine or special business as might come before them. These meetings took the place of the regular annual meeting for Indiana and Michigan, but Ohio will hold the regular annual meeting at the usual time and place, viz: in the first week of December next, and at Columbus.

One of the most enjoyable features of the occasion was the boat excursion up the Detroit River to the St. Clair Flats where a splendid entertainment was given at the Star Island House. Upon this occasion the Michigan Society was the host and the other State Societies, together with the visitors were the guests; and right royal was the entertainment from the moment of stepping on the boat till the return at eleven o'clock P. M. The occasion was one never to be forgotten by all who participated.

The success of the meeting as a whole was so pronounced and created such enthusiasm, that it was decided to hold another Tri-State meeting of the same Societies in 1898, and Committees were appointed to have the matter in charge.

Not the least important features of this meeting were its social advantages. Here were reunions of old or former acquaintances, and new acquaintance made that will be a joy for the future.

This meeting showed the importance and value of, at least, occasionally taking our Association work out of the routine groove in which it so generally runs, and projecting into it new features.

May not other States, that are favorably located, employ some such innovation, that will impart new energy and give new zest to dental society work. The social advantages are certainly worth more than such a meeting costs in time, effort and money.

To the local committee of arrangements of the recent Tri-State meeting at Detroit, viz: Drs. Geo. L. Feild, Joseph Lathrop and A. W. Diach, is due the thanks of all the participants of that grand occasion; for the credit is in a large measure theirs. Their efforts were unsparing to make the meeting what it was.

The thanks of the meeting was also due to the authorities of the Dental College for the excellent accommodation given; without charges, for all the purposes of the meetings, clinics, side meetings, etc.

